# Effect of Polylactic Acid (PLA) Blends on Cellulose Degradable Plastics from the Lotus Stem (*Nelumbo nucifera*)

**DOI:** 10.3390/polym17172281

**Published:** 2025-08-23

**Authors:** Rozanna Dewi, Novi Sylvia, Muhammad Subhan, Budhi Santri Kusuma, Aldila Ananda, Medyan Riza, Januar Parlaungan Siregar, Choon Kit Chan, Tezara Cionita, Elsherif Emad Ahmed Abdelrahman

**Affiliations:** 1 Chemical Engineering Department, Malikussaleh University, Lhokseumawe 24353, Aceh, Indonesia; novi.sylvia@unimal.ac.id; 2 Center of Excellence Technology Natural Polymer and Recycle Plastics, Malikussaleh University, Lhokseumawe 24353, Aceh, Indonesia; aldila.ananda12@gmail.com; 3 Department of Entrepreneurship, Malikussaleh University, Lhokseumawe 24353, Aceh, Indonesia; msubhan@unimal.ac.id; 4 Department of Industrial Engineering, University of Medan Area, Medan 20000, North Sumatra, Indonesia; budhi@staff.uma.ac.id; 5 Chemical Engineering Department, Syiah Kuala University, Banda Aceh 23111, Aceh, Indonesia; medyan_riza@usk.ac.id; 6 Faculty of Mechanical and Automotive Engineering Technology, Universiti Malaysia Pahang Al-Sultan Abdullah (UMPSA), Pekan 26600, Pahang, Malaysia; januar@umpsa.edu.my; 7 Centre for Automotive Engineering, Universiti Malaysia Pahang Al-Sultan Abdullah (UMPSA), Pekan 26600, Pahang, Malaysia; 8 Faculty of Engineering and Quantity Surveying, INTI International University, Seremban 71800, Negeri Sembilan, Malaysia; choonkit.chan@newinti.edu.my (C.K.C.); i18014237@student.newinti.edu.my (E.E.A.A.); 9 Faculty of Engineering, Built Environment & Information Technology, SEGi University, Petaling Jaya 47810, Selangor, Malaysia; tezara.cionita@newinti.edu.my

**Keywords:** green products, lotus stem, cellulose, mechanical, thermal, degradable plastic

## Abstract

Lotus stems contain cellulose, which can be utilized as a base material for producing green products, specifically degradable plastics. This research investigates the effect of polylactic acid (PLA) blends on cellulose degradable plastics from the lotus stem (*Nelumbo nucifera*). The mechanical characteristics are as follows: tensile strength of 0.7703–3.3212 MPa, elongation of 0.58–1.16%, Young’s modulus of 78.7894–364.6118 MPa. Compound analysis showed the presence of O-H, C-C, and C=O groups, and the presence of microbial activity in the soil can also lead to the degradation of these groups due to their hydrophilic nature, which allows them to bind water. Thermal analysis within a temperature range of 413.24 °C to 519.80 °C, shows that significant weight loss begins with the formation of crystalline structures. The degradable plastic exhibiting the lowest degree of swelling consists of 1 g of cellulose and 8 g of PLA, resulting in a swelling value of 6.25%. The degradable plastic is anticipated to decompose most rapidly after 52 days, utilizing 2 g of PLA and 7 g of cellulose. This complies with standard requirement, which sets a maximum degradation period of 180 days for polymers.

## 1. Introduction

In the last decade, there has been an almost explosive increase in the production and use of synthetic polymers, whose range of applications has revolutionized industries from packaging to automotives, healthcare, and electronics. As the world’s demand for plastics has increased, so has the amount of environmental pollution, especially that related to waste management and the non-biodegradability of traditional petroleum-based plastics [1]. In addition, progress towards sustainable urban environments is hampered by the lack of effective solutions for integrating modern technologies into industrial waste management [2]. The world is becoming more concerned about the environmental impact of oil-based plastics, and degradable plastics have appeared as one of the most promising solutions. Degradable plastics can be synthesized by utilizing various materials, including those derived from plant starch, cellulose, and waste materials. Making degradable plastic involves several stages, from selecting materials (such as plant starch and cellulose), to preparing them, and adding different ingredients. The goal is to alter the degradable plastic in some way to find optimal characteristics [3]. Producing polymers and nanocomposites involves using cellulose fibers, cellulose derivatives, and nanocellulose [4]. Natural fibers possess many advantageous properties, including low-density, suitable stiffness, and mechanical properties. In addition, plastic materials derived from natural fibers are recyclable and degradable [5]. Cellulose fibers can be used to produce films to increase their strength and durability [6]. Recent research has revealed the potential of plasticizers, mixing techniques, and the incorporation of cellulose-based products into degradable plastics as a viable and environmentally sustainable solution to address the limitations that are inherent to degradable plastics. The utilization of these materials, characterized by their abundance, biodegradability, and biocompatibility, holds promise in enhancing degradable plastics’ functionality and sustainability labels.

Biopolymers are a diverse class of macromolecules that are produced by microorganisms. In contrast, biopolymers are chemically synthesized from natural materials, such as polylactic acid (PLA). PLA has an increased tensile strength, which is defined as the maximum stress the polymer can withstand before it breaks under a tensile load, measured in MPa [7]. Nigam et al. [8] developed cellulose extraction from Parthenium hysterophorus weed as a base material for making bioplastics; the thermal stability of the extracted cellulose was found to be close to 350 °C and was used for bioplastics. Meanwhile, Yusoff et al. [9] demonstrated that a biocomposite made from starch and PLA had a significant increase in tensile strength with the addition of 30 wt.% tapioca starch, followed by a decline in tensile strength as the tapioca starch concentration increased. Lima et al. [10] introduced a technique for fabricating six biocomposites by extruding and injecting a blend of PLA matrix compositions and up to 20 wt.% mango seed by-products. Research from Arun et al. [11] suggests that composite-reinforced S. cumini biomass modified with polyvinyl chloride (PVC) can be a good material for medical devices, as it can resist bacterial contamination and address issues related to sustainability. Hemsri et al. [12] studied how PE-g-MA affects the structure, strength, and heat resistance of LDPE/poly(butylene adipate-co-terephthalate) (PBAT) films. The addition of a compatibilizer made the blends more uniform and improved their mechanical properties due to enhanced adhesion between the LDPE and PBAT phases. This change did not affect the thermal properties of the blends [12]. Ferreira et al. [13] studied the compatibility of PLA/BioPE with PE-g-MA. Compared to PLA, the binary blend exhibited reduced properties due to immiscibility. Incorporating PE-g-MA has been shown to promote interactions. Consequently, the properties of the materials exhibit enhanced mechanical and thermal stability [13].

The novelty of this innovation its utilization of cellulose fibers from lotus plants, namely the stem, and modifying them with PLA and a polypropylene graft maleic anhydride (PP-g-MA) compatibilizer. Several cellulose plants can be used as a base material for making degradable plastics, namely sugarcane bagasse, pineapple leaves, corn stalks, rice straw, etc. Lotus stems are often discarded as waste in lakes or incinerated in fields, resulting in ecological contamination and significant biomass resource wastage. Presently, investigation into lotus stem fiber remains in its early stages. The dearth of methodological research and critical performance metrics impedes the extensive implementation of lotus stem fiber [14]. Lotus stems containing cellulose fibers can be utilized more optimally because they harm esthetics and cause ecosystem disturbances, including flooding. The lotus stem (*Nelumbo nucifera*), commonly known as lotus, produces flowers and rhizomes with significant economic value. However, utilization of this vegetable is still limited. The edible parts of the plant are not limited to the flowers alone; the leaves, seeds, stems, and other parts can also be consumed and utilized. The analysis of the rhizome included the following nutrients: moisture (72.14%), starch (10.05%), carbohydrates (16.03 g), protein (2.6 g), fat (0.1 g), fiber (3.2 g), vitamin C (38 mg), calcium (40 mg), iron (1.07 mg), phosphorus (58 mg), and potassium (450 mg) [15].

The problem with degradable plastics is that their moldability is still low, making them difficult for industries to produce. Their strength is also not comparable to synthetic plastics, thus limiting their applications. Therefore, looking for innovations that can overcome these shortcomings is necessary. This research uses a new and innovative material, PLA, as a filler and PP-g-MA as a compatibilizer for degradable plastics made from lotus stem cellulose with thermopressing. Some researchers have used mineral fillers such as muscovite, tricalcium silicate, bentonite, calcite, and silicate to develop degradable plastics [16,17]. The incorporation of PLA into cellular-based degradable plastics has been identified as a strategy to enhance the optimal characteristics of degradable plastics, particularly their mechanical characteristics. PLA is a type of polymer that can decompose naturally [18,19].

In many cases, PLA is mixed with additional materials to improve its quality and characteristics. It can also be recycled, turned into compost, and made from renewable resources. PLA is the most advanced biopolymer available for commercial use, and it has been researched extensively [20]. PLA has a higher melting point of approximately 170–180 °C, which contributes to its advantageous properties. The glass transition temperature has been shown to increase to 58 °C. Higher temperatures at which a transition occurs, as well as higher melting points, are indicative of enhanced thermal stability in polymers [21]. Many studies have been published recently to improve the compatibility of the blend. PP-g-MA compatibilizer in degradable plastics can improve the morphology of the finely dispersed phase, enhance processability, and modify the crystallization behavior of the polyester component. These effects are attributed to an increase in phase interaction resulting in a reduction in interfacial tension [22]. In terms of its effectiveness as a compatibilizer in composite and polymer applications, PP-g-MA demonstrates notable performance [23].

This paper investigates the effect of polylactic acid (PLA) blends on cellulose degradable plastics from the lotus stem (*Nelumbo nucifera*). The variations in cellulose used in this study are 1, 3, 5, and 7 g, and the PLA variations are 2, 4, 6, and 8 g. The degradable plastic’s best characteristics were determined using cellulose weight variations.

## 2. Materials and Methods

### 2.1. Materials

The materials used in this study included lotus stems collected from the waters of Sawang, North Aceh, which were processed to obtain cellulose for producing degradable plastics. Other materials utilized included Xylene Merck (Supelco 108297, Burlington, MA, USA), Maleic Anhydride (MA) 99% (CAS No: 108-31-6, Shandong Arctic Chemical, Co., Ltd., Dongying, China), polypropylene (PP), Hydrogen Peroxide (H_2_O_2_) 30% 500 mL (SMARTLAB Merck, Jakarta Timur, Indonesia), Polylactic Acid (PLA) (C_3_H_6_O_3_) in 90.08 g/mol which was from Sigma-Aldrich–38534–1G, St. Louis, MO, USA, Polyethylene Glycol (CAS No: 25322-68-3, Fengyuan Chemical, Zaozhuang, China), Sodium Hydroxide (NaOH) (Merck-1310-73-2, Merck, Burlington, MA, USA), Sodium Hypochlorite (NaOCl) (Merck-2828 90 00, Merck, Burlington, MA, USA), and distilled water which was obtained from PT Bratachem, Surabaya, Indonesia.

### 2.2. Methods

The present research method comprises multiple stages, including the preparation of lotus stem powder, the extraction of cellulose, and the production and testing of degradable plastics. Table 1 shows the experimental design for the variable mass composition of lotus stem cellulose and PLA conducted in the study.

#### 2.2.1. Preparation of Lotus Stem Powder

Fresh lotus stems were thoroughly rinsed under running water to eliminate dirt and pollutants. The cleaned stems were segmented and sun-dried for one week to diminish their moisture content. The desiccated stems were subsequently severed into approximately 1 cm segments and pulverized into a fine powder utilizing a blender. The resultant powder was subjected to sieving using an 80-mesh screen to achieve a consistent particle size.

#### 2.2.2. Cellulose Extraction

Lotus stem powder was mixed with distilled water in a ratio of 1:10 (*w*/*v*) and stirred thoroughly. The mixture was heated to 100 °C for 60 min and subsequently filtered to isolate the solids. The solid residue was oven-dried at 105 °C before being submerged in 5% NaOH solution. The sample was then autoclaved at 121 °C for 60 min. Subsequently, 5% H_2_O_2_ was incorporated, and the mixture was subjected to reheating at 70 °C for 60 min. Thereafter, 10% NaOCl was added, and the mixture was heated for 30 min. The resultant material was meticulously cleaned with distilled water, oven-dried at 105 °C, and collected as alpha-cellulose powder.

#### 2.2.3. Synthesis of Degradable Plastics

A mixture consisting of 1 g of polylactic acid (PLA), 1 g of polyethylene glycol (PEG), polypropylene at 50% of the PLA weight, and 1 g of maleic anhydride was added into a beaker containing 10 mL of xylene. The mixture was subjected to heating and agitation at 125 °C to ensure the homogenous distribution of all constituents. At 150 °C, 1 g of the extracted cellulose was added and stirring continued until a uniform solution was achieved. The composite was then shaped using a hot press at 80 °C under a pressure of 130 kg/cm^2^ for a duration of 5 min. Subsequent to pressing, the samples were allowed to cool to ambient temperature. The resultant degradable plastics were further evaluated by mechanical, chemical, water absorption, and biodegradability tests to assess their qualities.

### 2.3. Characterization and Analysis

#### 2.3.1. Mechanical Properties

The ASTM D638 standard details the testing method for characterizing the tensile properties of plastic materials that have undergone either a reinforcement or no such process. This method is instrumental in determining critical mechanical properties, including tensile stress, strain, the elastic modulus, tensile strength, the tensile strength at yield, and the elongation at break. The mechanical properties were performed according to ASTM D-638-14 [24]. ASTM D638 Type I is the best specimen for rigid plastics shaped like a dumbbell. This specimen is 165 mm long, 3.2 mm thick (1/8 inch), and 50 mm (gauge length) (2 inches). This is similar to the thickness of common component materials. It ensures a good level of strain gauge measurement that is very accurate [24]. The sample formulations prepared and tested for mechanical analysis were lotus stem cellulose masses of 1, 3, 5, and 7 g, and PLA with a mass of 8 g, respectively.

#### 2.3.2. Chemical Properties

Identification of chemical compounds in diverse materials is facilitated by a technique known as Fourier transform infrared spectroscopy (FTIR), which employs the Shimadzu 8400S model (Shimadzu, Kyoto, Japan). This analytical instrument boasts a high degree of versatility, as it can measure the absorption and transmission of infrared radiation by a given sample. The resulting data provides insights into the molecular structure and composition of the material under investigation. In this chemical composition analysis, the sample formulation used was 7 g of lotus stem cellulose and 8 g of PLA.

#### 2.3.3. Thermal Properties

Thermal degradation is defined as the degradation of polymers caused by excessive heat. Hydrogen atoms are lost from the polymer chain during this process. The onset of thermal degradation determines the upper temperature limit for a polymer. Thermogravimetric analysis (TGA) is a technique used to measure changes in a material’s weight as it is heated or cooled, providing insights into the amount of mass lost during heating. This process can show how something changes when heated, such as how it dehydrates, decomposes, or oxidizes. The features of a thermogravimetric curve are determined by the properties of the material being studied and the chemical compound because of the typical order of physical and chemical reactions that happen within a specific temperature range and heating rate. The evaluation of thermal stability for degradable plastics is typically conducted using TGA (Model TGA50 SrC30025100553), New Orleans, LA, USA. In this analysis, the sample formulation was 7 g of lotus stem cellulose and 8 g of PLA.

#### 2.3.4. Water Absorption

The water resistance of degradable plastics is analyzed through water absorption testing. The swelling is determined using a standard procedure (ASTM D2765) [25]. The first step is to weigh the samples and soak them in solvent for 24 h. After the initial swelling, the samples undergo a re-weighing and drying process. The sample formulations prepared and analyzed for water absorption by swelling test were lotus stem cellulose (1, 3, 5, 7 g), which were examined using various PLA amounts (2, 4, 6, 8 g); more details can be seen in Table 1. The swelling degree is calculated using Equation (1).

(1) $$Swelling=\frac{Weight\;of\;expanded\;sample-Weight\;of\;pre\;sample}{Pre-weight\;sample}\times 100\%$$

#### 2.3.5. Biodegradability Rate

The burial-in-soil test was performed to assess the material’s biodegradability, in accordance with ASTM G-21-13 [26]. This process involves deterioration by direct contact between the degradable plastic and the soil. Plastic specimens measuring 5 cm × 2 cm were fabricated, weighed, and subsequently interred at a depth of 30 cm. Following a three-day period, the samples were collected, cleaned, and reweighed to ascertain the mass loss. The samples were prepared and analyzed to see how quickly they break down. The temperature and pH levels used in biodegradability analysis (32 °C and 5.5–7) are widely considered to be suitable to facilitate biodegradation. However, it should be noted that the specific parameters for soil moisture required during the burial process for biodegradable plastics depend on the prevailing environmental conditions. Lotus stem cellulose (1, 3, 5, 7 g) was examined using various PLA amounts (2, 4, 6, 8 g); see Table 1 for more details. Finally, the biodegradability of the plastic was calculated using Equation (2).

(2) $$\mathrm{Biodegradability}\;(\%)=\frac{M_{0}-M_{1}}{M_{0}}\times 100\%$$

where *M*_0_ is the pre-mass, or the initial weight, in grams. *M*_1_ is the final mass, the weight at the end, in grams.

## 3. Results

### 3.1. Mechanical Properties Result

**Figure 1:**
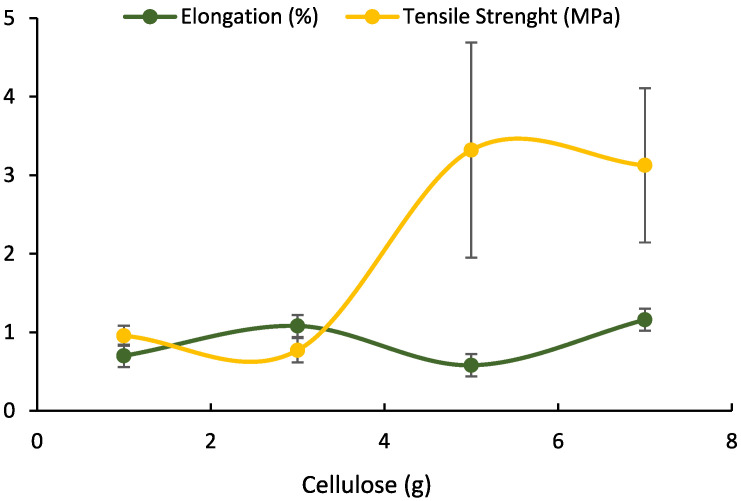
Mechanical properties of degradable plastics from variable amounts of lotus stem cellulose–PLA.

**Figure 2:**
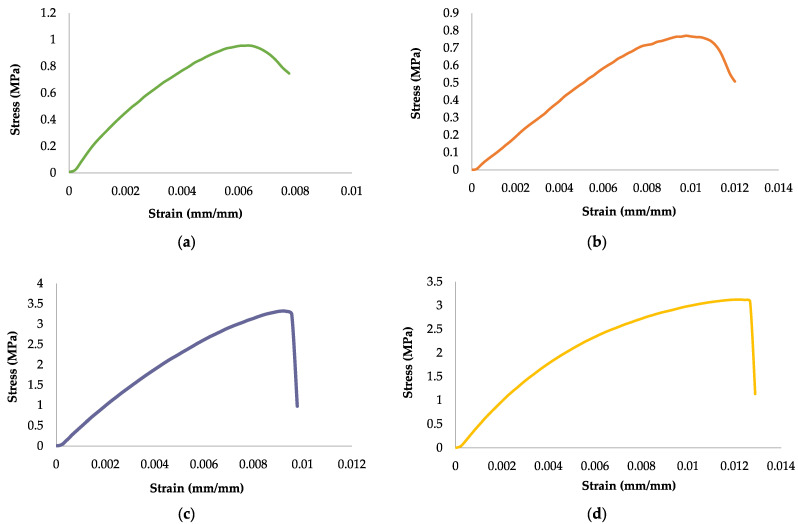
Stress–strain curves of degradable plastic with variations in lotus stem cellulose: (**a**) 1 g of cellulose, (**b**) 3 g of cellulose, (**c**) 5 g of cellulose, and (**d**) 7 g of cellulose.

In this research, a texture analysis tool was used to determine the material’s tensile strength, measuring its stiffness and ability to stretch. The tensile strength is optimized, allowing it to resist external forces before the degradable plastic undergoes deformation or rupture. The occurrence of breakage is possible due to cracking resulting from over-stress or structural deformation. The tensile strength test results shown in Table 2 and Figure 1 were obtained with a weight variation in lotus stem cellulose mass (1, 3, 5, and 7 g) and 8 g of PLA mass. Figure 2a–d, show the stress–strain curves of degradable plastic with variations in lotus stem cellulose.

### 3.2. Chemical Properties Result

**Figure 3:**
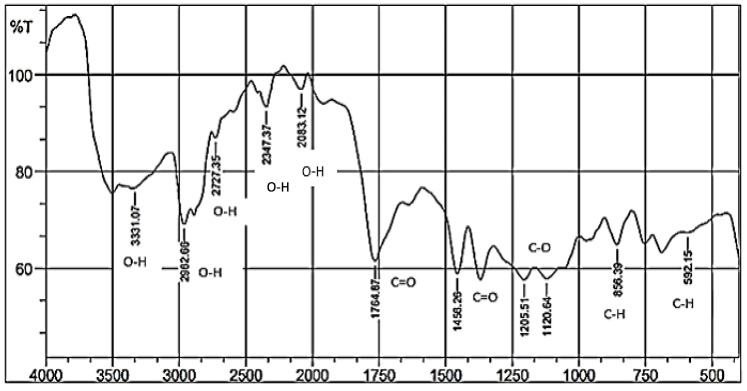
FTIR analysis of degradable plastic made from lotus stem cellulose–PLA.

In this study, Fourier-transform infrared spectroscopy (FTIR) was used to determine the composition of the materials. The molecular structure of the sample determines the wavelength that it absorbs (Table 3). FTIR was conducted at a range of wave numbers from 550 to 4000 cm^−1^ with 7 g of lotus stem cellulose and 8 g of PLA (Figure 3).

### 3.3. Thermal Properties Result

**Figure 4:**
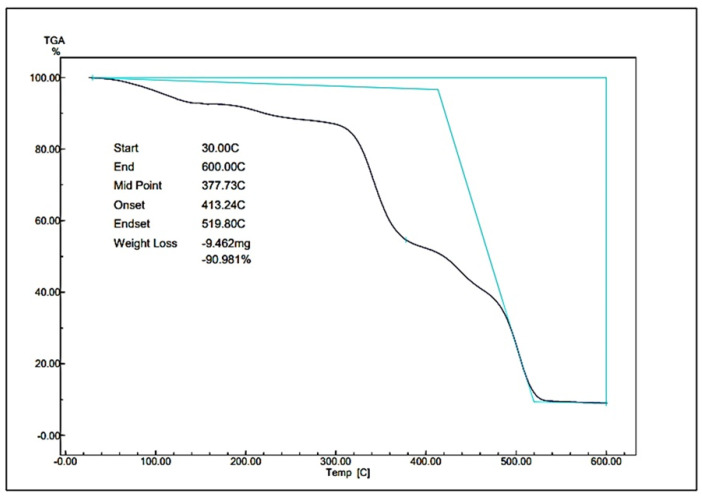
TGA of lotus stem cellulose–PLA degradable plastic.

The thermal stability of the degradable plastics was assessed by thermogravimetric analysis (TGA), which quantifies mass variations in response to temperature elevation and can indicate physical transitions such as glass transition and melting. The test was conducted on biodegradable plastic comprising 7 g of cellulose and 8 g of PLA. The TGA curve presented in Figure 4 depicts the variation in mass as a function of temperature.

### 3.4. Water Absorption Result

**Figure 5:**
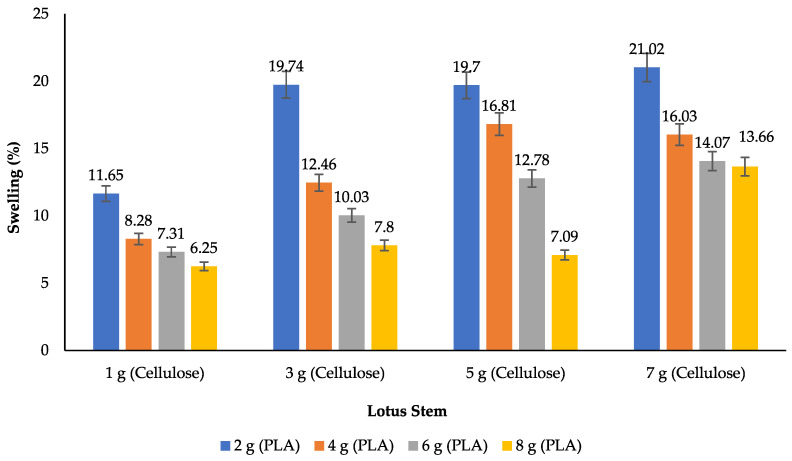
Water absorption of degradable plastic with variations in lotus stem cellulose–PLA.

Water absorption indicates the process by which bioplastics absorb moisture. Figure 5 illustrates the water absorption data of the lotus stem degradable plastic: lotus stem cellulose (1, 3, 5, 7 g) was examined using various PLA amounts (2, 4, 6, 8 g) in the swelling tests.

### 3.5. Biodegradability Rate with Soil Burial Result

**Figure 6:**
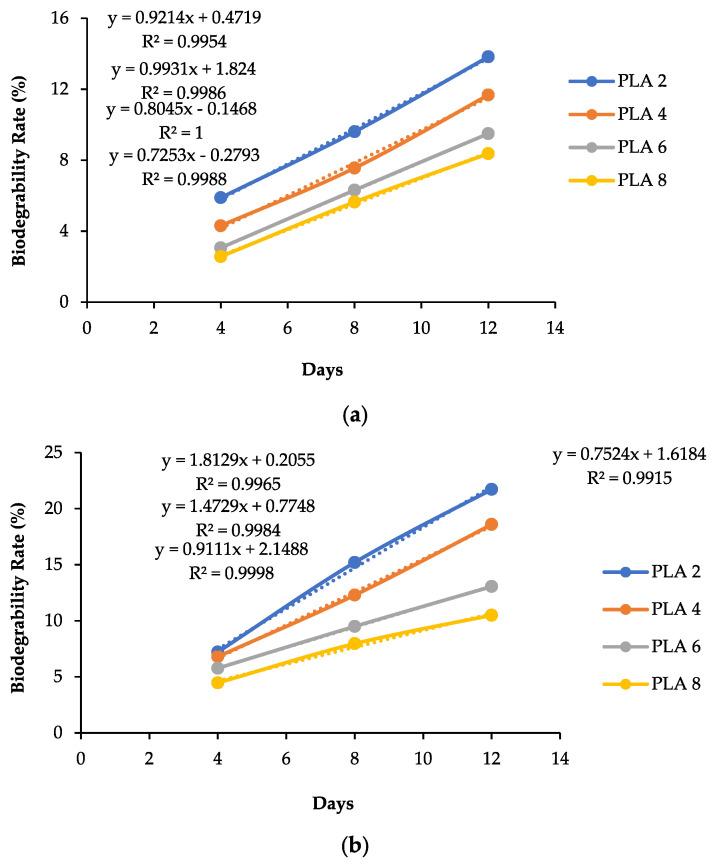

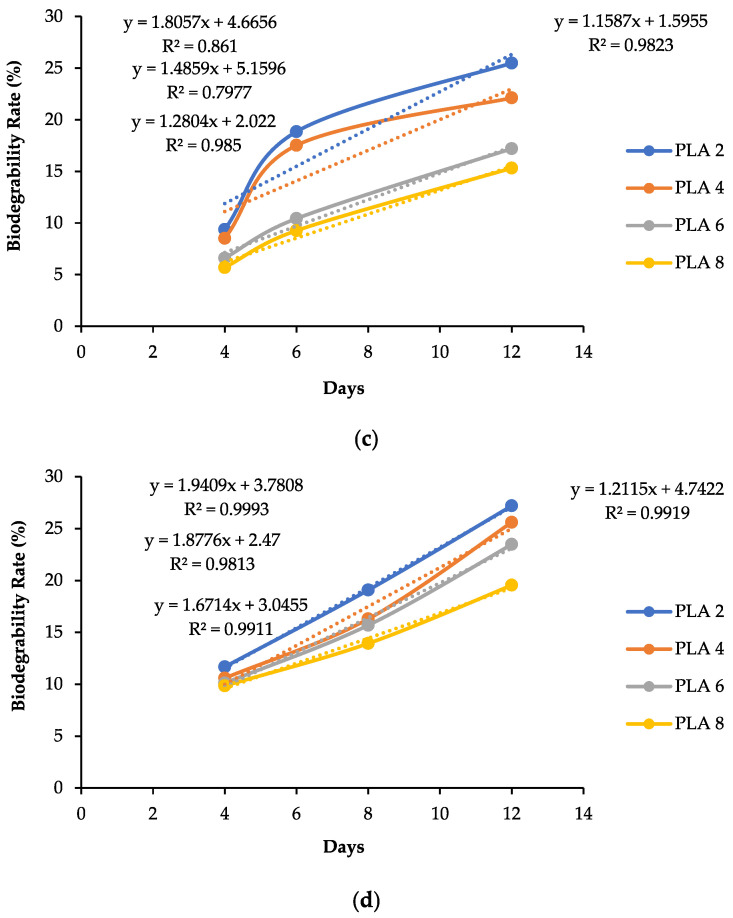
Biodegradability rate of lotus stem degradable plastic with variations in PLA and cellulose amounts: (**a**) 1 g of cellulose, (**b**) 3 g of cellulose, (**c**) 5 g of cellulose, and (**d**) 7 g of cellulose.

Biodegradation testing is a method used to determine the decomposition rate of biodegradable plastics. This process aims to determine the time required for the plastic in question to undergo complete decomposition within a given soil environment. As shown in Table 4 and Figure 6, the biodegradability analysis results of the lotus stem degradable plastic with various lotus stem cellulose amounts (1, 3, 5, 7 g) were examined with various PLA amounts (2, 4, 6, 8 g) in the soil burial tests.

## 4. Discussion

### 4.1. Mechanical Properties Analysis

An investigation was conducted into the mechanical properties of degradable plastics to ascertain their respective tensile strengths, elongation, and Young’s modulus values. The findings indicate that the cellulose composition of each type of degradable plastic studied is generally homogeneous, as demonstrated by the minimal standard deviation values observed in the tensile strength and elongation curves depicted in Table 2 and Figure 1. Figure 2 illustrates the relationship between stress-and-strain curves, indicating no statistically significant difference. Table 2 indicates that for degradable plastics incorporating different masses of lotus stem cellulose (1, 3, 5, and 7 g) with a fixed PLA content of 8 g, the tensile strengths obtained were 0.9561 MPa, 0.7703 MPa, 3.3212 MPa, and 3.1258 MPa, respectively, with the peak value observed at 5 g of cellulose. These results show that, with natural fiber cellulose, degradable plastics become more resistant. It can be seen that the more cellulose added, the greater the tensile strength obtained. The incorporation of polylactic acid (PLA) has been demonstrated to enhance the tensile strength of the resulting plastic. The tensile strength value was shown to be unstable, possibly due to the stirring process of materials that were not well mixed. Uneven stirring of the material causes the mixing of the material to be non-homogeneous, resulting in an imperfect degradable plastic, which will affect the tensile strength of the degradable plastic [27]. If the filler is not physically held in place, it might move out of the matrix when loaded. This can make it harder for stress to move efficiently and can reduce the strength of the material. The strength of the material can be increased even more by creating chemical bonds linking the filler and the matrix [28].

A recent study showed that incorporating 2.0 wt.% of a silicate–glycerol mixture into a sago starch/polyvinyl alcohol (PVA) film enhanced its tensile strength and flexibility compared to films without the silicate addition [29]. Cao et al. [30] created an enhanced paper film using response surface methodology (RSM) with a Box–Behnken design, incorporating a mixture of cow dung and hemp fiber. The resultant material demonstrated a tensile strength of around 8.26 MPa and a tear strength of approximately 19.91 N/mm. Werchefani et al. [31] utilized an alpha fiber and PLA matrix for biocomposites with the injection molding method, and the mechanical strength of the composites increased by 17% and 45%, respectively, when 20% alpha fiber was treated with NaOH. The study indicates a need for further research to optimize the mechanical performance. However, when compared to the tensile strength of pure PLA in Table 1, the use of 5 g and 7 g of cellulose mass is almost comparable [27].

The measurement of a material’s capability to exhibit tensile strain under the application of a tensile force is denoted as its elongation. This is an important property that determines how a material responds to pressure. Degradable plastics are more elastic, so the material can be easily bent before breaking. Elasticity, defined as the ability of a material to stretch, is a crucial factor in the analysis of materials science. The material property known as Young’s modulus can quantify a material’s elasticity and its ability to resist pressure. In the hypothesis that the force ceases, the object will revert to its original state. It is imperative to note that the ratio of stress to strain remains constant. It has been established that an increase in the modulus of elasticity corresponds to a decrease in the material’s degree of deformability, leading to a greater degree of rigidity [32]. According to Singh et al. (2020) [33], the rigidity and strength of the material were higher than those of PLA/poly(ethylene oxide) and similar to pure PLA. However, the material’s ability to stretch before it breaks was 73–143% higher because poly(ethylene oxide) and cellulose fiber effectively changed its properties and made it stronger [33].

Table 2 shows that the elongation of degradable plastics with variations in lotus stem cellulose mass (1, 3, 5, and 7 g) with a PLA mass of 8 g is 0.70–1.16%, while the Young’s modulus obtained is 78.7894–364.6118 MPa. According to the Mat Web Material Property Database, the category “Polypropylene, Extrusion Grade” has an elongation at break of 8–750% and a Young’s modulus of 0.680–3.60 GPa. The comparison of elongation and the modulus of elasticity reveals that these materials are not directly similar to polymers produced from lotus stems. The tensile strength of pure PLA varies from 5.00 to 42.0 MPa, elongation varies from 15.0% to 100%, and Young’s modulus varies from 2960 to 3600 MPa—metrics that remain substantially different from those of the lotus stem-based samples. This study indicates that filler density is the primary factor affecting mechanical properties, as its augmentation correlates directly with enhanced mechanical strength. The improvement is likely attributable to stronger adhesion between the PLA matrix and the fiber. Meanwhile, the work carried out by Wojciechowska [34] revealed that the elongation at break shows an increasing trend with increasing process temperature. Conversely, at a reduced temperature, the molecules within the polymer matrix exhibit indications of solidification, accompanied by minimal energy dissipation. According to Lin et al. [35], PP-g-MAH can only enhance the interfacial adhesion between glass fiber (GF) and polypropylene (PP), but not the deformation resistance. Additionally, an increase in the concentration of PP-g-MAH results in a decrease in molecular weight. Natural fibers have unique advantages, but they also have limitations. One of their limitations is that they do not stick well to polymer matrices. One of the methods to deal with this problem is to use coupling agents [35]. Studies conducted by Yaghoobi and Fereidoon [36] have shown that PP-g-MA improves the adhesion between the fibers and the matrix. The molecular weight significantly impacted the tensile and flexural strengths, resulting in either the maintenance of these values or a decline. According to the extant literature, elongation measurement at break increased with a decrease in molecular weight [37].

### 4.2. Chemical Properties Analysis

As shown in Figure 1, the sodium hydroxide compound in cellulose exhibits OH stretching vibrations by wave numbers of 3331.07 cm^−1^, 2962.66 cm^−1^, 2727.35 cm^−1^, 2347.37 cm^−1^, and 2083.12 cm^−1^. The lower wave number shows a decrease in the OH strain peak. Furthermore, there is a C-H bond, an aromatic compound in the wave range of 592.15 cm^−1^ and 856.39 cm^−1^. Razali et al. [38] conducted a study on the pulping of cellulose fibers from rice straw as a polymer base material. The study revealed the presence of typical cellulose bands, including OH stretching at wave numbers of 3327 cm^−1^. The cellulose–PLA blends break down because of changes in the hydrogen bonds in the material. This is shown by decreased O-H stretching in the earlier FTIR analysis [39]. Wave numbers of 1764.87 cm^−1^, 1456.26 cm^−1^, 1205.51 cm^−1^, and 1120.64 cm^−1^ are identified as C=O and C-O stretching vibrations present in xylene and PP-g-MA compositions, with the potential to be associated with PLA. The carbonyl group (C=O) causes the degradation of polypropylene (PP) used in degradable plastics. Carbonyl (C-O) formation has been identified as an indicator of polyethylene degradation [38]. Researchers calculated the carbonyl index to study the degradation of recyclable low-density polyethylene (LDPE), as outlined in the extant literature. The index is determined by comparing the strength of the carbonyl band at 1715 cm^−1^ to the strength of the methylene bond at 1464 cm^−1^, as determined by established criteria [40]. The presence of specific functional groups has been shown to influence the interface binding of starch and MA molecules in degradable plastics. This interaction could potentially affect these materials’ mechanical and physical properties [20].

In addition, Gbadeyan et al. [41] utilized a combination of snail shell and bagasse cellulose to produce bioplastics, employing polylactic acid (PLA) as a synthetic polymer in FTIR analysis. The FTIR findings exhibited a significant presence of C-O, C=O, and -CH stretching vibrations of -COOH functional groups at the interface. This indicated the presence of functional groups within the bioplastic films [41]. The cluster content is an organic compound in the FTIR study of degradable plastic made from lotus stem cellulose. This finding suggests the polymer exhibits hydrophilic properties, facilitating its binding to water. This phenomenon precipitates its facile degradation by soil. It also shows more free hydroxyl groups (-OH) in the degradable plastic. This is because fewer molecules can bond with hydrogen. Sodium hydroxide (NaOH) or other extractives provide the -OH groups [32].

### 4.3. Thermal Properties Analysis

As illustrated in Figure 2, the TGA graph of lotus stem cellulose-and-PLA-based degradable plastic is presented. Weight loss was observed in the degradable plastic sample. The findings indicated that cellulose bioplastics exhibited satisfactory thermal stability at elevated temperatures. The initial stage of degradation of cellulose degradable plastics occurred at an approximate temperature of 30–377.73 °C. At the second stage, the onset of extreme weight loss, characterized by the formation of crystalline structures, occurs within the temperature range of 413.24 °C to 519.80 °C. The study found that the plastic made from lotus stem and PLA degradable materials lost 90.981% of its weight. This result indicates that 9.462 mg of the plastic material remained. It has been determined that changes in the thermogram of TGA result from two factors: first, the transfer of heat from the degradable plastic; and second, the following investigation examines the structural and phase alterations in degradable plastic. The material undergoes substantial decomposition at this stage, decomposing completely at 600 °C. The study demonstrated that the degradable plastic exhibited significant thermal degradation. The samples showed signs of losing hydrogen groups, decomposing, depolymerizing, and cracking carbon chains in the cellulose structure [42,43].

Dewi et al. [27] investigated the thermal properties by TGA on degradable plastics from PLA-modified avocado seed starch, losing weight slowly starting from 30 °C, Tmax at 356.86 °C to 413.64 °C, and a residual mass of 97.500% with 4.290 mg remaining. On the other hand, Gunti et al. [44] revealed that in biocomposites including cellulose fibers and PLA, degradation began at 322 °C and was completed within the temperature range of 322–430 °C. The cellulose-derived biodegradable plastics decomposed at a lower temperature than pure cellulose, a phenomenon that is attributable to their amorphous structure. Cellulose decomposes into char, a solid carbon-rich residue composed of polycyclic aromatic compounds, within the temperature range of 370–600 °C [45]. In previous studies, the strength of nanocomposites was found to increase in comparison to that of pure PLA, particularly at a high temperature of 45 °C, which exhibited a 50% increase in strength. It has been determined that there is an absence of detrimental impacts on the molar mass of the PLA matrix caused by standard fibers and nanofibers. Additionally, the weight of composite materials, including nanocomposites, remained constant up to a temperature of 300 °C [46]. A correlation has been demonstrated between the residual mass after decomposition and the thermal resistance of materials such as lotus stem cellulose-and-PLA-based degradable plastics. The findings indicate that the greater the residual weight following decomposition is, the more superior the thermal resistance of the material under consideration is.

Oliver et al. [47] studied how bio-based polymers, like thermoplastic starch (TPS) and polylactic acid (PLA), break down when heated in a nitrogen atmosphere. The study found that using different starch types and calcium carbonate additives in TPS can improve how well these materials resist high temperatures, which is vital for future industrial uses. Zor et al. [48] studied biomass-based nanocomposites and found that as the amount of nano lignin increased, there was less mass loss and a lower decomposition temperature. Studies show that treating fiber-reinforced polymer composites with chemicals can significantly change their thermal degradation behavior (TGA), which measures how quickly a material loses mass when heated [48]. This treatment can also impact the thermal stability of the composite structure, meaning how well it can resist heat damage [49]. PLA’s degradation temperature (225 °C) exceeds its melting temperature (170 °C). This makes it worthwhile in polymer and composite materials, such as injection molding. Other bioplastics start degrading at a lower temperature after melting, which limits the processing temperature range [50].

### 4.4. Water Absorption Analysis

The degradation of degradable materials is contingent upon their absorption of water, which serves as the medium through which they undergo decomposition. The microbial growth and consumption process is initiated once these materials have absorbed water, thereby serving as a source of energy for the microorganisms involved [29]. Figure 3 presents the swelling values of lotus stem degradable plastic with a PLA amount of 2 g (11.65–21.02%), 4 g (8.28–16.03%), 6 g (7.31–14.07%), and 8 g (6.25–13.66%). The degradable plastic with the smallest swelling degree consists of 1 g of cellulose and 8 g of PLA, with a value of 6.25%. Meanwhile, the degradable plastic with the highest water absorption properties is found in that with 7 g of cellulose and 3 g of PLA content, with a value of 21.02%. This indicates that the greater the amount of PLA used and the less cellulose used, the better the degradable plastic is because it has the smallest percentage value of water absorption [27]. Chemical changes are the best way to improve how well PLA and cellulose interact. Two main chemical changes make cellulose’s surface less water-repelling and make PLA molecules more hydrophilic. Cellulose has many hydroxyl groups on its surface, which make it hydrophilic [51]. Polymeric materials such as PLA exhibit complex and hydrophobic properties, rendering them compatible with a wide range of products [52]. Soil burial tests revealed that the biodegradability of the degradable plastic increased with increasing cellulose addition, as the water absorption of the degradable plastic directly influenced the biodegradability. Water absorption tests also showed that biocomposites with low water resistance increased the degradation rates of the PLA–cellulose blends [53].

The integration of fillers in the manufacturing process of degradable plastics is attributable to three key factors: the inherent characteristics of renewable materials, the advantageous properties of biodegradability, and the substantial availability of such materials. Incorporating fillers into degradable plastics has improved a range of mechanical properties, including the rigidity, tensile strength, barrier properties against gas, melting point, and heat stability. Modifying sago starch plastic films reduces their water absorption capacity, thereby rendering them more hydrophobic [16]. This phenomenon can be attributed to substituting hydrogen bonds in the starch molecular structure with phosphate groups. The result is a film with increased resistance to water. Pure PLA exhibits a water absorption rate of 0.3%, leading to a 0.66% swelling in width [54]. Based on the results, water absorption was strongly influenced by the interfacial compatibility and structure of PLA/cellulose. PP-g-MA compatibilizer helps reduce the amount of water absorbed by the material’s surface when it is used. Poor compatibility can cause spaces and paths for water to enter into the material, which increases how much water it absorbs [32].

### 4.5. Biodegradability Rate with Soil Burial Analysis

Biodegradability testing is performed to determine how long each sample degrades. The way to determine the biodegradability rate in samples is by using extrapolation calculations to estimate the value of a variable beyond the original observation interval based on its relationship with other variables. The extrapolation formula of lotus stem degradable plastics with variations in cellulose mass is shown in Figure 6a–d.

Table 3 shows that all the degradable plastics underwent weight changes with different percentages. After 12 days of embedding, the data shows a considerable mass reduction rate in the degradable plastic sample with 7 g of cellulose and 2 g of PLA at 27.19%. From Figure 6a–d, it can be seen that the degradable plastics are expected to degrade completely within 52–100 days. The sample with 7 g of cellulose and 2 g of PLA exhibited the highest degradation rate, with complete degradation estimated at 52 days. At the same time, the longest estimated degradation at 100 days was shown with a PLA content of 8 g and 1 g of cellulose. Amaba et al. (2023) [55] used the casting method to determine the effect of three main components on the biodegradability of bioplastics, namely chitosan, glycerol, and starch. The resulting bioplastics showed optimal biodegradability [55]. Dewi et al. [27] found that avocado starch-based degradable plastic with the addition of PLA resulted in a biodegradability rate of 37.988% for 12 days. Meanwhile, Brunšek et al. [56] investigated bioplastics composed of cellulose fibers and PLA and discovered that flax fibers demonstrated the most mass loss after 11 days of microbial exposure. Jute and sisal fibers exhibited significant losses, with jute losing 44.51% of its mass and sisal losing 7.92%. The findings indicate that the chemical composition of the fibers significantly influences their degradation rate.

ASTM D-6002 is a guide for checking if environmentally degradable plastics can be composted. The ASTM D-6002 standard explains what makes a plastic degradable [57]. The composition of polymers, whether they are homopolymers or random copolymers, necessitates a minimum organic carbon content of at least 60%. This means that they must turn at least 60% of the carbon into carbon dioxide within 180 days. The dosage of the filler material type exerted a significant influence on the composite’s overall biodegradability properties [58]. The composite developed by Rasheed et al. (2021) using CNC (natural fiber) and PLA-PBS (degradable polymer) is very important because it can completely break down in soil, which makes it an environmentally friendly alternative to existing packaging materials [59]. Pure PLA does not absorb much water because it is hydrophobic. This research explains how PLA biodegrades and absorbs water in Macadamia seeds and PLA biocomposites. It also shows how using natural resources in composite materials can affect sustainability [60]. The use of synthetic materials also significantly improves the results of bioplastics. However, the biodegradability of reinforced bioplastics differs based on how well the synthetic and bioplastic materials interact [61,62]. Plastic based on PLA, about a quarter of all bio-based plastics, can break down naturally over time. The review of the literature on PLA-based plastics shows that scientists understand how these plastics break down in both aerobic and anaerobic environments [63]. Also, optimization of wear behavior from PLA is possible by incorporating microcrystalline cellulose [64].

## 5. Conclusions

In conclusion, the mechanical properties test showed that 5 g of cellulose combined with 8 g of PLA created the strongest material, measuring at 3.3212 MPa, while a material made of 7 g of cellulose had a slightly weaker result at 3.1258 MPa. The tensile strength of pure PLA was between 5.00 MPa and 42.0 MPa, showing that both materials performed similarly in terms of strength. The highest elongation value, or the amount of stretch before it breaks, was found in the 7 g of cellulose sample at 1.16%. The pure PLA sample, which has a range of 15.0% to 100% for this property, could not be compared to the cellulose sample. The tensile strength value of the plastic tends to increase with the addition of PLA. However, the tensile strength value was found to be unstable, possibly due to the mixing process of materials that were not thoroughly mixed. The cluster content seen in the FTIR analysis of degradation-resistant polymers derived from lotus stem cellulose is a group of molecules that are connected by covalent bonds. This means that the plastic can react with water and break down easily in the soil. In TGA, research has shown that the amount of material left over after it has been decomposed is related to how well certain materials can resist heat, like lotus stem cellulose-and-PLA-based degradable plastics. The findings show that if a material remains heavier after degradation, the thermal resistance of the material is higher. The degradable plastic with the smallest swelling degree was that with the composition of 1 g of cellulose and 8 g of PLA with a value of 6.25%. The degradable plastic is estimated to degrade the fastest at 52 days, namely with a PLA content of 2 g with 7 g of cellulose. The longest estimated degradation at 100 days is that with a PLA content of 8 g with 1 g of cellulose.

Lotus stems contain cellulose fibers and need to be utilized more optimally to increase their added value because currently their use in superior products is still limited, mostly left unattended, and detrimental especially in terms of esthetics. Lotus is considered an invasive species, which means that it can grow and proliferate in unwanted areas, often causing disruption to the surrounding ecosystem. The degradable plastics in this paper are an attractive alternative to conventional petroleum-based plastics due to their nature that is inclusive and the potential environmental benefits they offer. To achieve comparable mechanical characteristics to those of commercial and conventional polymers, there is a necessity to enhance the processing technology of producing degradable plastics from lotus stem cellulose and PLA. The utilization of degradable plastics in the field of product manufacturing has become increasingly relevant, as these materials possess inherent stability in their thermal properties, rendering them suitable for various applications, such as their use in household products, interior design elements, and in numerous other items.

## Figures and Tables

**Table 1:** Experimental design for variable mass composition of lotus stem cellulose and PLA.

Lotus Stem Cellulose Mass (g)	PLA Mass (g)
1	2
1	4
1	6
1	8
3	2
3	4
3	6
3	8
5	2
5	4
5	6
5	8
7	2
7	4
7	6
7	8

**Table 2:** Mechanical properties of degradable plastics from variable amounts of lotus stem cellulose–PLA.

Cellulose Mass (g)	PLA Mass (g)	Tensile Strength (MPa)	Elongation (%)	Young’s Modulus (MPa)	Standard Deviation Tensile Strength-Elongation
1	8	0.9561	0.70	153.6248	0.12805
3	8	0.7703	1.08	78.7894	0.15485
5	8	3.3212	0.58	364.6118	1.3706
7	8	3.1258	1.16	255.7704	0.9829
0	Pure PLA	5.00–42.0	15.0–100	2960–3600	-

Matweb (material property data)—pure PLA.

**Table 3:** FTIR spectrum of degradable plastic made from lotus stem cellulose and PLA.

Bonding	Compound Type	Wave Numbers (cm^−1^)	Peak
O-H	Alcohol Monomer	3331.07, 2962.66, 2727.35, 2347.37, 2083.12	Sharp and Witdh
C-H	Aromatic	592.15, 856.39	Width
C=O	Carbonyl	1764.87, 1456.26	Sharp
C-O	Alkena	1205.51, 1120.64	Sharp

**Table 4:** Biodegradability analysis of degradable plastic made from lotus stem cellulose and PLA.

Cellulose Mass (g)	PLA Mass (g)	Biodegradability Analysis (%)		
		4	8	12
1	2	5.882	9.598	13.827
	4	4.302	7.553	11.673
	6	3.059	6.312	9.494
	8	2.564	5.639	8.366
3	2	7.210	15.202	21.713
	4	6.802	12.287	18.585
	6	5.765	9.493	13.053
	8	4.467	7.960	10.486
5	2	8.958	18.850	25.495
	4	7.692	17.531	22.125
	6	5.097	10.433	17.204
	8	4.674	9.267	15.320
7	2	10.367	19.070	27.190
	4	9.981	16.294	25.600
	6	8.493	15.686	23.467
	8	7.013	13.927	19.533

## Data Availability

The original contributions presented in this study are included in the article. Further inquiries can be directed to the corresponding author.
